# Disseminated cutaneous Herpes Simplex Virus-1 in a woman with rheumatoid arthritis receiving Infliximab: A case report

**DOI:** 10.1186/1752-1947-2-282

**Published:** 2008-08-26

**Authors:** Elizabeth Ann Justice, Sophia Yasmin Khan, Sarah Logan, Paresh Jobanputra

**Affiliations:** 1 Rheumatology Department, Selly Oak Hospital, University Hospital Birmingham NHS Trust, Raddlebarn Road, Birmingham, B29 6JD, UK

## Abstract

**Introduction:**

We present the case of a 49-year-old woman with a seronegative rheumatoid arthritis who developed pustular psoriasis whilst on etanercept and subsequently developed disseminated herpes simplex on infliximab.

**Case presentation:**

Our patient presented with an inflammatory arthritis which failed to respond to both methotrexate and leflunomide, and sulphasalazine treatment led to side effects. She was started on etanercept but after 8 months of treatment developed scaly pustular lesions on her palms and soles typical of pustular psoriasis. Following the discontinuation of etanercept, our patient required high doses of oral prednisolone to control her inflammatory arthritis. A second biologic agent, infliximab, was introduced in addition to low-dose methotrexate and 15 mg of oral prednisolone. However, after just 3 infusions of infliximab, she was admitted to hospital with a fever, widespread itchy vesicular rash and worsening inflammatory arthritis. Fluid from skin vesicles examined by polymerase chain reaction showed Herpes Simplex Virus type 1. Blood cultures were negative and her chest X-ray was normal. Her infliximab was discontinued and she was started on acyclovir, 800 mg five times daily for 2 weeks. She made a good recovery with improvement in her skin within 48 hours.

She continued for 2 months on a prophylactic dose of 400 mg bd. Her rheumatoid arthritis became increasingly active and a decision was made to introduce adalimumab alongside acyclovir. Acyclovir prophylaxis has been continued but the dose tapered so that she is taking only 200 mg of acyclovir on alternate days. There has been no recurrence of Herpes Simplex Virus lesions despite increasing adalimumab to 40 mg weekly 3 months after starting treatment.

**Conclusion:**

We believe this to be the first reported case of widespread cutaneous Herpes Simplex Virus type 1 infection following treatment with infliximab. We discuss the clinical manifestations of Herpes Simplex Virus infections with particular emphasis on the immunosuppressed patient and the use of prophylactic acyclovir. Pustular psoriasis is now a well recognised but uncommon side effect of antitumour necrosis factor therapy and can lead to cessation of therapy, as in our patient's case.

## Introduction and Case presentation

**Figure 1:**
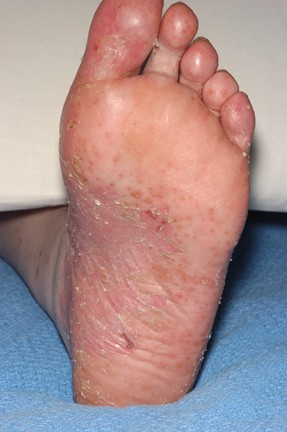
**Scaly pustular lesions on soles of feet typical of pustular psoriasis**.

**Figure 2:**
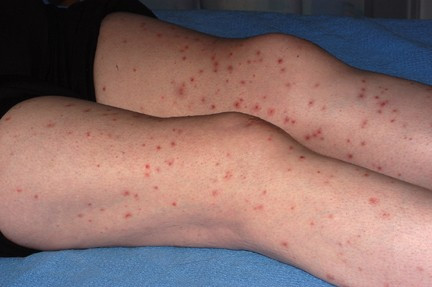
Vesicular rash on lower legs.

We describe a 49-year-old woman with seronegative polyarthritis who developed pustular psoriasis whilst on etanercept and subsequently developed disseminated herpes simplex on infliximab in combination with methotrexate. Our patient presented in 2004 and was initially treated with methotrexate. She was unable to tolerate doses beyond 15 mg per week because of troublesome mouth ulcers. Her disease failed to come under control and she was dependent on oral prednisolone at doses above 20 mg. After 5 months, she was switched to sulphasalazine 3 g daily. She developed severe headaches and 3 months later was switched to leflunomide 20 mg daily without any clinical improvement. Her erythrocyte sedimentation rate (ESR) was raised at 44 mm/hour despite oral prednisolone at 25 mg daily and 10 months after diagnosis was started on Etanercept 25 mg subcutaneous injections twice weekly combined with low-dose oral methotrexate (10 mg/week). Three months later, her ESR had fallen to 26 mm/hour and the oral prednisolone reduced to 10 mg daily. Eight months after starting etanercept, she developed scaly pustular lesions on her palms and soles typical of pustular psoriasis (Fig. 1). She ceased etanercept temporarily and her skin improved markedly but her arthritis worsened. On restarting etanercept, the pustular psoriasis recurred. She switched to infliximab, administered intravenously at a dose of 3 mg/kg, and she received the first 3 infusions over the course of 6 weeks. Three weeks after her third infusion, she was admitted to hospital with a fever, a widespread vesicular rash (Fig. 2) and a flare of her arthritis. On admission, she was taking prednisolone 15 mg daily and methotrexate 5 mg weekly. Her full blood count revealed a total count of 17.5 × 10^9 ^with a neutrophilia of 10.9 × 10^9^, a C-reactive protein of 153 mg/litre, and an ESR of 75 mm/hour. Renal and liver function tests were normal and immunoglobulins A, G and M were normal. There was no growth on blood cultures and her chest X-ray (CXR) was unremarkable. Fluid from skin vesicles examined by polymerase chain reaction showed Herpes Simplex Virus type 1 (HSV-1). Serological tests showed no evidence of acute Varicella Zoster Virus but indicated past exposure. Infliximab was discontinued and acyclovir 800 mg five times daily was given for 2 weeks. She improved systemically and her vesicular rash started to resolve within 48 hours of acyclovir.

The dose of acyclovir was then reduced to 400 mg twice daily and after 2 months on this dose, she commenced a third anti-TNF agent adalimumab, 40 mg subcutaneous injections once a fortnight. Acyclovir prophylaxis has been continued, so far, for 8 months but the dose tapered so that she is taking only 200 mg of acyclovir on alternate days. There has been no recurrence of HSV-1 lesions despite increasing adalimumab to 40 mg weekly 3 months after starting treatment. Her current dose of prednisolone is 10 mg od.

## Discussion

We believe that this is the first description of widespread cutaneous HSV-1 infection following treatment with infliximab. Our patient also developed pustular psoriasis whilst taking etanercept and the psoriasis worsened on re-challenge with etanercept.

HSV-1 is one of the ubiquitous herpes family of viruses usually transmitted during childhood. Around 60% of adults show evidence of past infection and the primary infection is often mild or asymptomatic [1]. Reactivation of the virus following latency in the sensory ganglia can happen years later and manifest usually as cold sores. Characteristically painful vesicles develop in a localised area such as the lip which subsequently progress over days to non-scarring scabs. The extent of cutaneous disease is determined by a number of host factors including age, intercurrent illness, immune status and presence of pre-existing skin disease. Disseminated cutaneous disease may occur in immunocompromised patients, especially those with haematological malignancies and following bone marrow and organ transplants. Encephalitis [2], hepatitis [3] and pneumonia [4] caused by HSV are also more common in immunocompromised patients.

We are not aware of any published reports of serious HSV infections associated with use of TNF inhibitors and cannot say whether treatment with infliximab, steroids alone, or the drug combination caused disseminated HSV-1 in our patient. *In vivo *data indicate that TNF-alpha may have an antiviral effect in HSV-1 infections. In a model in which HSV-1 was reactivated in latently infected mice cornea, TNF-α and interleukin-6 were the predominant cytokines within the trigeminal ganglion suggesting a key role for these cytokines in viral clearance [5]. Absence of TNF in knockout mice increased susceptibility to primary corneal HSV-1 infections in one study [6] and lowered survival rates compared with wild-type mice in another (83% cf 97%) [7]. Whilst all three TNF inhibitors used in clinical practice inhibit the actions of TNF-α, their different mechanisms of action may result in a variable susceptibility to HSV-1 infections, although this has not specifically been studied.

*In vitro *studies of gingival fibroblasts showed that cells pretreated with dexamethasone and infected with HSV-1 gave rise to higher yields of virus [8] suggesting that corticosteroids increase susceptibility to infection in these cells. Recipients of renal transplants on high doses of prednisolone (above 25 mg daily) are reported to have twice the rate of HSV infections compared with those on lower doses including primary infections in seronegative patients and re-infections of seropositive patients [9]. Unfortunately, this study does not report the severity and nature of HSV-1 infections seen.

In several small placebo-controlled trials, prophylactic use of oral acyclovir in immunocompromised patients has been found to be successful in reducing the duration of viral shedding and preventing clinical HSV infections in 80% to 100% of patients [10]. The oral doses studied were 200 mg tds for 30 days and 200 mg qds for 180 days. In both studies, there were no additional adverse events compared with placebo. The most frequently reported adverse effects during acyclovir therapy are headache, nausea and abdominal cramping. Whilst oral acyclovir has a good safety profile, cases of rapidly progressive acute neurological and renal toxicity have been described [11]. Acyclovir-induced neurotoxicity can present with a variety of symptoms including agitation, delirium and hallucinations [12]. Dose reductions are recommended in patients with renal impairment and in the elderly. Our patient received 400 mg twice a day for 4 months and has since reduced the dose to 200 mg alternate daily. Whilst current evidence around the optimum duration and dose for long-term prophylaxis is lacking, a decision to continue on this low level of therapy was taken with the patient because of concerns about infection recurrence. Once acyclovir therapy is discontinued, there is no ongoing protection against HSV infections.

Pustular psoriasis and psoriasis are an uncommon but recognized adverse event associated with TNF-α inhibition. The British Society for Rheumatology Biologics Register reported that, among 8672 patients with rheumatoid arthritis treated with anti-TNF therapy, there were 23 reports of a new onset of psoriasis in patients with no previous history of psoriasis and one patient with a family history of psoriasis. Eight of the 23 patients stopped treatment and of those, six reported an improvement in their psoriasis. Overall psoriasis rates were over four times higher in patients treated with TNF-α inhibitors compared with patients treated with other disease modifying antirheumatic drugs [13]. One large case series of 13 patients included only one case induced specifically by etanercept [14].

There is no clear explanation as to the nature of this phenomenon. Sfikakis *et al. *postulate that under certain conditions, TNF-α inhibition promotes the activation of autoreactive T cells leading to tissue damage via autoimmune pathways [15] whilst De Gannes *et al. *speculate that increased expression of interferon-α in the dermal vasculature may increase susceptibility to psoriatic skin lesions [14].

## Conclusion

This is the first reported case of disseminated cutaneous HSV-1 infection following treatment with infliximab in a patient with rheumatoid arthritis. We believe this unusual adverse reaction to be as a direct result of her immunosuppressive therapy.

Prophylactic acyclovir may reduce the frequency and severity of recurrent HSV attacks although the exact dose and duration of therapy are uncertain and likely to vary according to individual circumstances.

Our patient had also developed pustular psoriasis whilst on etanercept. Psoriatic skin reactions are a recognised but uncommon side effect of anti-TNF therapy and may require cessation of treatment.

## Competing interests

Elizabeth Justice, Sophia Khan and Sarah Logan declare that they have no competing interests.

Dr Paresh Jobanputra has been involved in commercially sponsored trials of adalimumab and etanercept in rheumatic diseases. He has also received support for educational purposes from Wyeth and Abbott Laboratories.

## Authors' contributions

EAJ and SJK prepared the manuscript and performed the literature search. SL participated in data collection. PJ approved the final manuscript. All authors read and approved the final manuscript.

## Consent

Written informed consent was obtained from the patient for publication of this case report and any accompanying images. A copy of the written consent is available for review by the Editor-in-Chief of this journal.
